# PB-Motif—A Method for Identifying Gene/Pseudogene Rearrangements With Long Reads: An Application to CYP21A2 Genotyping

**DOI:** 10.3389/fgene.2021.716586

**Published:** 2021-07-28

**Authors:** Zachary Stephens, Dragana Milosevic, Benjamin Kipp, Stefan Grebe, Ravishankar K. Iyer, Jean-Pierre A. Kocher

**Affiliations:** ^1^Department of Electrical and Computer Engineering, University of Illinois Urbana-Champaign, Urbana, IL, United States; ^2^Mayo Clinic, Rochester, MN, United States

**Keywords:** long reads, pseudogene, structural variation, congenital adrenal hyperplasia, CYP21A2, bioinformatics, computational biology

## Abstract

Long read sequencing technologies have the potential to accurately detect and phase variation in genomic regions that are difficult to fully characterize with conventional short read methods. These difficult to sequence regions include several clinically relevant genes with highly homologous pseudogenes, many of which are prone to gene conversions or other types of complex structural rearrangements. We present PB-Motif, a new method for identifying rearrangements between two highly homologous genomic regions using PacBio long reads. PB-Motif leverages clustering and filtering techniques to efficiently report rearrangements in the presence of sequencing errors and other systematic artifacts. Supporting reads for each high-confidence rearrangement can then be used for copy number estimation and phased variant calling. First, we demonstrate PB-Motif's accuracy with simulated sequence rearrangements of *PMS2* and its pseudogene *PMS2CL* using simulated reads sweeping over a range of sequencing error rates. We then apply PB-Motif to 26 clinical samples, characterizing *CYP21A2* and its pseudogene *CYP21A1P* as part of a diagnostic assay for congenital adrenal hyperplasia. We successfully identify damaging variation and patient carrier status concordant with clinical diagnosis obtained from multiplex ligation-dependent amplification (MLPA) and Sanger sequencing. The source code is available at: github.com/zstephens/pb-motif.

## 1. Introduction

Next-generation sequencing technologies have become ubiquitous in a wide range of diagnostic assays at many clinical laboratories. Targeted capture of gene regions, whole exome sequencing, and whole genome sequencing are increasingly used for patient genotyping. While conventional short read platforms are the most widely used for reporting clinically relevant genetic variation, they are poorly equipped to characterize regions of the genome with low complexity, large repeated elements, or with structural organization significantly different from that of the reference genome (Lee and Schatz, 2012; Mandelker et al., 2016).

Many genes are difficult to characterize due to the presence of highly homologous pseudogenes. Pseudogenes are nonfunctional genomic DNA sequences with high similarity to functional genes, often originating from retrotransposition of mRNA or from ancestral duplications of functional genes (Bischof et al., 2006). Proximal gene/pseudogene pairs are of particular interest because rearrangements between the two regions, typically from unequal crossing over or gene conversion events, can render the gene nonfunctional (Bischof et al., 2006). This mechanism has been shown to be a driver in many diseases, including Lynch syndrome (van der Klift et al., 2010), Hunter syndrome (Zhang et al., 2011), chronic granulomatous disease (Moens et al., 2014), among others (Bischof et al., 2006; Sen and Ghosh, 2013).

Studies in genome “mappability” have highlighted the difficulty of uniquely aligning short reads to different genes (Derrien et al., 2012; Lee and Schatz, 2012; Li et al., 2014; Stephens and Iyer, 2018). This challenge has been mostly overcome with the development of longer read sequencing platforms, such as those from Oxford Nanopore or PacBio's Single Molecule Real Time (SMRT) technologies. These platforms are capable of generating reads long enough to uniquely map many gene/pseudogene regions. Despite this, comprehensive genotyping (including variant phasing and copy number estimation) is still complex and labor intensive due to the increased frequency of structural rearrangements in these regions (Sen et al., 2010). Conventional variant and structural variant calling workflows often perform poorly in these regions, with multi-mapped and mismapped reads hindering the detection of breakpoints and variant sites. This challenge is further complicated by copy number variation and gene/pseudogene chimeras which are difficult to accurately align in the presence of sequencing errors.

To address this challenge, we present PB-Motif, a new methodology leveraging long reads for the *de novo* identification of arbitrary structural rearrangements confined to a pair of genomic regions. PB-Motif is applicable to PacBio long reads from targeted sequencing, e.g., by capture-probes, PCR, or other methods to selectively enrich regions of interest. We demonstrate PB-Motif's effectiveness on both simulated data and real clinical samples. We use PB-Motif as part of a diagnostic assay for 21-hydroxylase-deficient congenital adrenal hyperplasia (21-OHD CAH) using PacBio long reads. Specifically, we sequenced 26 samples and reported structural rearrangements and small variants known to affect *CYP21A2* function. The copy number estimates and phased variants were concordant with results from multiplex ligation-dependent amplification (MLPA) and Sanger sequencing. Based on these results, we believe this PB-Motif could be widely applied to many clinically relevant gene/pseudogene pairs in the human genome that cannot be easily characterized with short reads.

## 2. Materials and Methods

**Figure 1:**
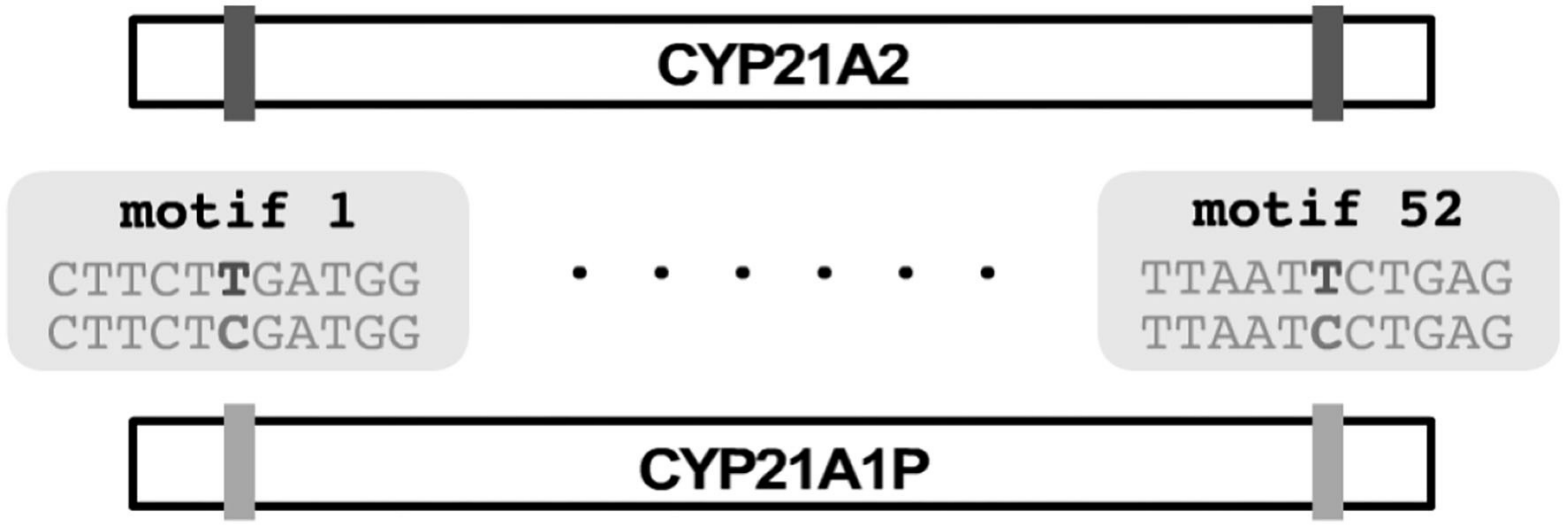
PB-Motif uses gene and pseudogene “motifs” which are kmers centered on sites which differentiate gene and pseudogene reference sequences when aligned to each other.

**Figure 2:**
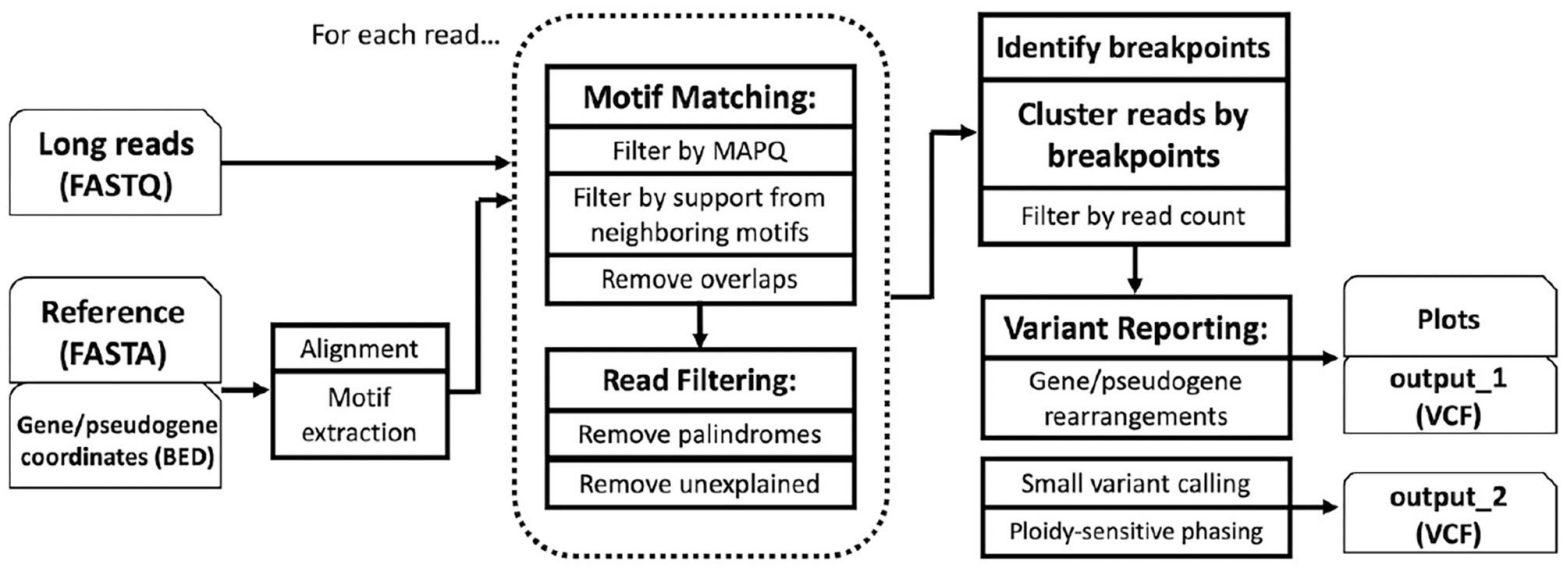
PB-Motif overview.

PB-Motif leverages polymorphic mutations in pseudogenes to discriminate between gene and pseudogene sequences and to infer structural reorganizations between these regions, if present. To begin, PB-Motif aligns gene and pseudogene reference sequences against each other, enumerating kmers of a specified size (default: 11) which are found in one region but not the other. These kmers are centered on nucleotides which differentiate gene and pseudogene at given positions, and anchored on either side by sequence which is identical is both regions. We refer to kmers that are unique to the gene (i.e., do not occur in the pseudogene) as *gene motifs*, and conversely kmers unique to pseudogene are referred to as *pseudogene motifs* (Figure 1). PB-Motif takes as input a set of gene/pseudogene motifs alongside long reads (in FASTQ format) sequenced from the corresponding regions (Figure 2).

###  Finding Motifs in Long Reads

We define *G* = [*g*_1_, *g*_2_, …, *g*_*N*_] and *P* = [*p*_1_, *p*_2_, …, *p*_*N*_] as the sequence of gene and pseudogene motifs, respectively, sorted by genomic coordinate. We define *Ĝ* = [*ĝ*_1_, *ĝ*_2_, …, *ĝ*_*N*_] and $\hat{P}=\left[{\hat{p}}_{1},{\hat{p}}_{2},\ldots ,{\hat{p}}_{N}\right]$ as the positions where these motifs occur in the reference sequence.

To begin, each read is scanned for exact matches to all motifs in *G* and *P*, with the requirement that all matching positions are above a specified base-call quality score (default: 10). Next, we require that multiple consecutive motifs are found in groups of 2 or greater. For example, if *g*_*n*_ is found in a read, it will only be retained if either *g*_*n*−1_ or *g*_*n*+1_ are also found within the same read. Further, we require that the expected distance between consecutive motifs differs by no more than *D* from their distance in the reference genome (default: 10). For example, if *g*_1_ and *g*_2_ were found at read coordinates ${\hat{r}}_{1}$ and ${\hat{r}}_{2}$, they would be discarded as errors if $|\left({\hat{r}}_{2}-{\hat{r}}_{1}\right)-\left({\hat{g}}_{2}-{\hat{g}}_{1}\right)|>D$. We justify these constraints with the assumption that structural rearrangements between a gene and its pseudogene will involve enough sequence to span multiple motif sites. That is, we choose not to interpret an isolated pseudogene-derived variant in an otherwise unaltered gene to be a consequence of a structural rearrangement.

Motif matches are often confounded by sequencing errors or genetic variants, preventing them from being detected via an exact search. In an effort to recover motifs missed due to random errors, we allow the motif group requirement described above to be loosened by a user-specified parameter *M*: If a motif *g*_*n*_ is found, but neither *g*_*n*−1_ or *g*_*n*+1_ are present, we continue searching up to *g*_*n*±*M*_. For example, if a sample's genome had motifs *g*_1_, *g*_2_, *g*_3_ consecutively, but a particular read sequenced has a base-call error that prevents *g*_2_ from being found, *g*_1_ and *g*_3_ would be retained as a motif group for *M* ≥ 2.

All groups of motifs are then tested for overlap with each other in read coordinates, and if a conflict is found (i.e., a particular span of read coordinates supports multiple motif groups) we apply a graph search approach similar to our previous methodology (Stephens et al., 2018), discarding groups that do not belong to the highest scoring path of motifs through the read.

###  Additional Read Filtering

Amplicon-based long reads are known to be prone to certain artifacts (Smyth et al., 2010; Laver et al., 2016), including palindromic reads, off-target reads, and *in-vitro* PCR recombination (also called PCR chimerism).

Palindrome artifacts result from PacBio circular reads where adapter sequences are not properly identified and the sequence string in the FASTQ contains both the forward and reverse strand of the template sequence (appended to each other, separated by an adapter sequence). While there exist tools to prune these reads (Warris et al., 2018), we found it equally effective and less computationally intensive to implement a filter in PB-Motif directly. Each read is tested by aligning its sequence of detected motifs against itself backwards. Specifically, a read is discarded if >50% of its length is comprised of motif groups that align to their reverse complement. In our applications this filter typically removes 1–2% of the total reads.

Off-target reads, despite not originating from the gene or pseudogene regions of interest, may contain spurious motif matches that pass initial detection filters. Under the assumption that such reads will likely contain large spans of sequence where no motifs are found, we implement a filter to discard reads if the amount of sequence unexplained by known gene or pseudogene motifs exceeds a user-specified portion of its length (default: 30%).

###  Identifying and Clustering Breakpoints

If the sequence of motifs found in a read differ from their expected order in the reference genome, they provide evidence for potential structural rearrangements. Mixtures of gene and pseudogene motifs in a the same read provide evidence for junctions between the two regions. We notate each read as a sequence of observed motifs *R* = [*r*_1_, *r*_2_, …, *r*_*n*_], *r*_*i*_ ∈ *G* ∪ *P*, with read coordinates $\hat{R}=\left[{\hat{r}}_{1},{\hat{r}}_{2},\ldots ,{\hat{r}}_{n}\right],{\hat{r}}_{i}\in \left[0,l_{r}-1\right]$ where *l*_*r*_ is the length of the read.

For each read *R*, we define *t*(*R*) = [*t*_1_, *t*_2_, …] to be the sequence of tuples representing the starting and ending reference coordinates of all contiguous motif groups in the read. A contiguous motif group is a subsequence of motifs which all belong to the same set (*G* or *P*) and are strictly increasing by no more than *M*. As an example, consider a hypothetical read *R* = [*g*_1_, *g*_2_, *g*_3_, *g*_1_, *g*_2_, *g*_3_, *p*_8_, *p*_9_, *p*_10_] which has three contiguous motif groups: [*g*_1_, *g*_2_, *g*_3_], [*g*_1_, *g*_2_, *g*_3_], and [*p*_8_, *p*_9_, *p*_10_]. In this case $t\left(R\right)=\left[\left({\hat{g}}_{1},{\hat{g}}_{3}\right),\left({\hat{g}}_{1},{\hat{g}}_{3}\right),\left({\hat{p}}_{8},{\hat{p}}_{10}\right)\right]$.

PB-Motif's clustering step begins by first grouping reads with identical motif sequences, producing a weight matrix *W*_1 × *N*_ specifying the number each reads supporting each of the *N* observed sequences. Next we apply hierarchal clustering using the distance function:

$$D(R_{i},R_{j})=\{\begin{array}{ll} \sum_{n=1}^{\left|t(R_{i})\right|}{||t{\left(R_{i}\right)}_{n}-t{\left(R_{j}\right)}_{n}||}_{1} & \left|t(R_{i})|=|t(R_{j})\right|\\ \infty & \left|t(R_{i})|\neq |t(R_{j})\right| \end{array}$$

This distance function is 0 for reads with identical motif sequences, infinite for reads with differing numbers of motif groups, and finite for reads with the same number of groups (but the groups themselves are not identical). With this we compute the distance between all *N* observed motif sequences, yielding a pairwise distance matrix *D*_*N*×*N*_.

With *W* and *D* we perform greedy intermediate-linkage clustering (Algorithm 1). In practice this strategy tends to produce a limited number of clusters supported by a large number of reads, and many weakly-supported clusters corresponding to artifacts such as PCR chimeras or off-target reads. This algorithm has two parameters: α, the maximum tolerated distance a read can be from a candidate cluster, and β, the proportion of reads in an existing cluster that need to be close enough to a new read for it to be added. By default PB-Motif uses α = 10 and β = 0.5.

**Algorithm 1:**
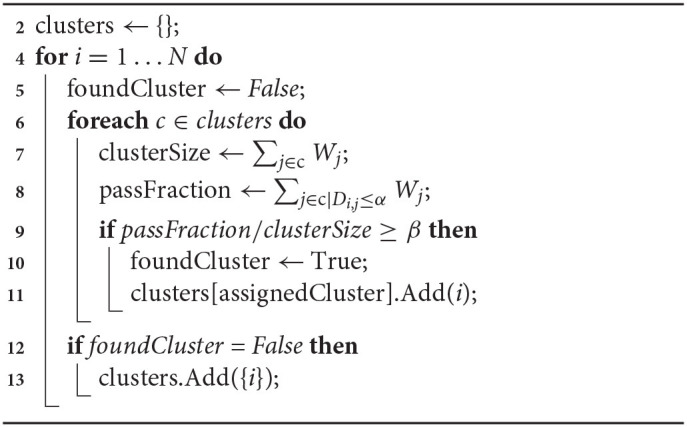
Greedy intermediate-linkage clustering

###  Variant Calling and Annotation

For clinical CAH samples, we annotate rearrangements reported by PB-Motif based on their impact on *CYP21A2* function. Specifically, we bin read clusters into five categories based on their configuration of gene and pseudogene sequences:

- Normal *CYP21A2* sequence
- Normal *CYP21A1P* sequence
- A1P → A2 (e.g., chimeras resulting from unequal crossing over)
- A2 → A1P (e.g., results of gene conversion)
- Other (e.g., rearrangements with >2 breakpoints).

Copy numbers for each cluster are estimated using read count proportions and variant allele frequencies. For our 26 clinical samples, copy numbers were validated using MLPA (Supplementary Figure 1).

Small variants are called on clusters of reads corresponding to normal *CYP21A2* sequence using a genotyping workflow with minimap2 (Li, 2018) and Mutect2 (DePristo et al., 2011; Cibulskis et al., 2013). Mutect2 was chosen because the copy number in each cluster is unknown *apriori*, and thus we cannot use a variant caller that requires ploidy to be specified at runtime. Variants known to be associated with CAH are annotated using variant lists extracted from existing CAH literature (Simonetti et al., 2018).

In inherited diseases like CAH variant phasing is crucial to determining carrier status if multiple damaging variants are present. As a final step we phase damaging variation using a clustering methodology similar to the popular tool Whatshap (Patterson et al., 2015). Given *m* reads and *n* variant sites, we construct an allele matrix *A*_*m*×*x*_ by locally realigning reads around each variant site and choosing the allele to which the read sequence aligns with the lowest edit distance. Hierarchal clustering is performed using Ward's method on the pairwise hamming distance between all reads. The dendrogram is then cut at a user-specified value and the phased variants from each cluster are written to an output VCF file.

###  Sample Selection

In total we selected 26 samples from our clinical labs spanning a range of CAH phenotypes. All analysis was performed on deidentified DNA under the approval of Mayo Clinic Institutional Review Board (Application number 21-002875). Samples were selected predominantly on the basis of whether we believed variant phasing and copy number estimations from long reads would supplement existing MLPA and Sanger results to help resolve ambiguous or challenging cases. As such, we expect our cohort to be enriched for samples with unusual genotypes.

###  Sequencing

The traditional methodology for 21-OHD CAH genotyping uses combinations of primers specific to the 5′ and 3′ ends of *CYP21A1P* and *CYP21A2*, with Sanger sequencing and MLPA for variant calling and copy number assessment, respectively (Greene et al., 2014; Kluge et al., 2020). However, this approach is complex and labor intensive, yields ambiguous results in some cases, and does not provide information on variant phasing across an entire gene, which is informative in assessing carrier status.

Our sequencing approach uses a single primer pair for amplifying *CYP21A2* and *CYP21A1P* simultaneously. Specifically, we use primers placed in the promoter and 3′ tail regions that flank both *CYP21A2* and *CYP21A1P* (Table 1, Supplementary Figure 2). The resulting PCR yields a mixture of amplicons that include (i) normal gene sequence, (ii) normal pseudogene sequence, (iii) any sequence that begins in the gene and ends in the pseudogene, and (iv) any sequence that begins in the pseudogene and ends in the gene. The amplicons underwent a second round of PCR, which utilized a universal priming site introduced in the first PCR, to add a barcoded adapter sequence (16 bp) that uniquely labeled the sample and allowed for multiplexing during sequencing. Samples were then pooled together in equal mass, and a SMRTbell library was prepared for sequencing on a PacBio Sequel using the SMRTbell Template Prep Kit 1.0, following manufacturer protocol. Sequencing of the primer binding and polymerase annealing were done using the Sequel Binding Kit 2.1 and sequencing primer v3 in accordance to instructions provided in SMRTlink 7.0. An overview of SMRTbell sequencing is presented in Travers et al. (2010) and Rhoads and Au (2015). The Circular Consensus Sequences application was used to generate FASTQ data (referred to as “HiFi” reads) which averaged 3.7 kb in length and had minimum predicted accuracy of 90%.

**Table 1:** Forward and reverse primer sequences for amplicon sequencing of *CYP21A2* and *CYP21A1P*.

Forward	5^′^ CAGAAAGCTGACTCTGGATGCAGG 3^′^
Reverse	3^′^ AACTGCCACTACGCCAACCTCAAC 5^′^

## 3. Results

###  Simulated Data

**Figure 3:**
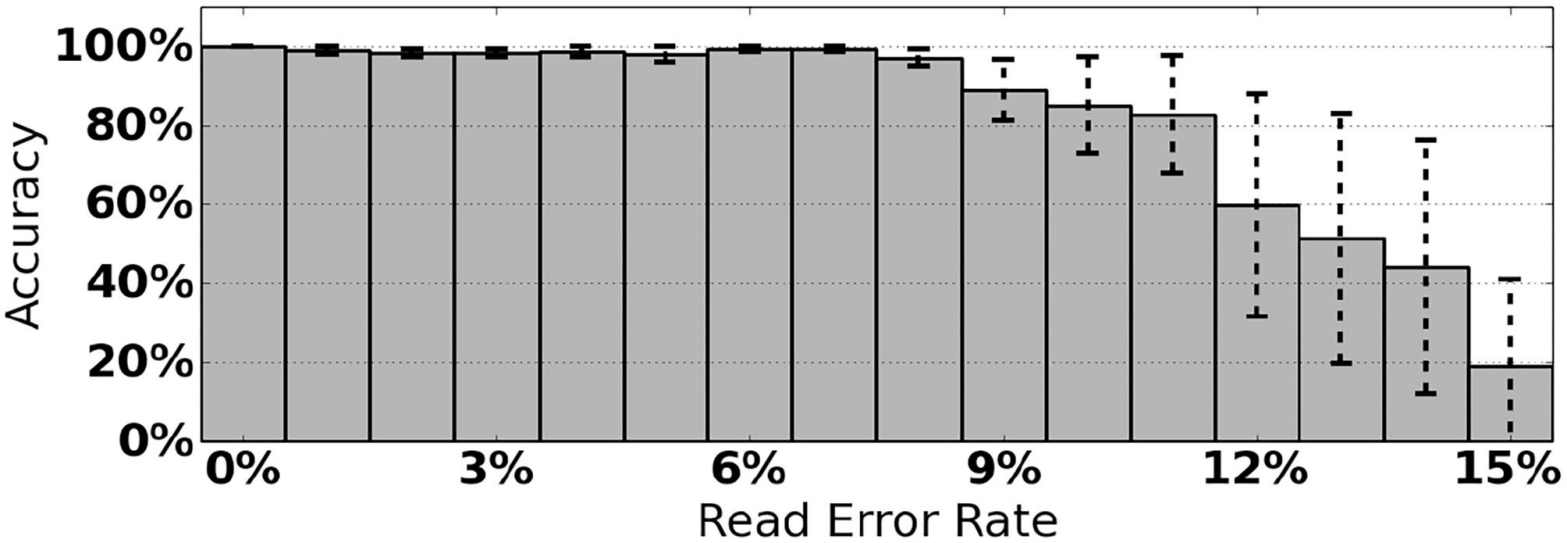
Mean and standard deviation detection accuracy for simulated *PMS2*/*PMS2CL* rearrangements.

As an initial assessment of PB-Motif, we analyze synthetic data where ground truth rearrangements are known a priori. The purpose for this is two-fold: (i) to identify read error rates at which PB-Motif can no longer confidently identify rearrangements, even under ideal conditions, and (ii) to demonstrate PB-Motif's theoretical extensibility to gene/pseudogene pairs beyond *CYP21A2*/*CYP21A1P*. To do this we generate synthetic datasets containing rearrangements of *PMS2* and its pseudogene *PMS2CL*. We enumerate 30 motifs across a 6 kbp homology and generate synthetic PacBio reads spanning this region using the NEAT simulator (Stephens et al., 2016). We imputed five classes of rearrangements: Deletions, gene/pseudogene chimeras, pseudogene/gene chimeras, tandem duplications, and dispersed duplications. Read error rates were swept from 0 to 15% and each simulation was replicated 10 times, resulting in a total of 800 synthetic datasets. Each dataset was then processed by PB-Motif, and accuracy was computed as the proportion of reads supporting the correct simulated rearrangement (Figure 3).

###  Clinical CAH Samples

We processed 26 CAH samples with PB-Motif and reported gene/pseudogene rearrangements and SNVs (Table 2). Rearrangements and small variants were phased for each sample, but we report aggregated genotypes for each sample group (CAH, NCCAH, and carriers) in compliance with deidentification requirements. Variant calling was performed on clusters of reads corresponding to normal gene sequence (or rearranged sequences that are comprised mostly normal gene sequence), because it is in these clusters that the presence of damaging variation could render a gene nonfunctional and thus be of clinical interest. The reported genotypes were compared against results from MLPA and Sanger sequencing as well as clinical notes accompanying each sample, which generally included physical examination and 17α-Hydroxyprogesterone (17-OHP) measurements. Specifically, copy numbers for normal gene, normal pseudogene, and chimeras were compared against MLPA (example shown in Supplementary Figure 3), and variation in gene regions were validated with Sanger sequencing (example shown in Supplementary Figure 4). Small variant calling was restricted to damaging variants only (as identified by existing CAH literature; Simonetti et al., 2018), and every variant presented in Table 2 was confirmed by Sanger.

**Table 2:** Summary of genotypes for SW CAH, NCCAH, and carrier sample groups.

	**CYP21A2 copy number**	**A1P/A2 chimeras**	**A2/A1P conv.**	**Damaging variation**
SW & SV CAH samples (10)	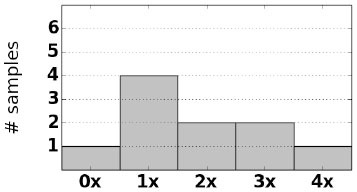	1 × CH1	1	g.-9C>T
		3 × CH2		g.655C>G
		3 × CH3		g.707_714del (p.Gly110ValfsX21)
		1 × CH5		g.999T>A (p.Ile173Asn)
		1 × CH7		g.1994C>T (p.Gln318X)
				g.2578C>T (p.Pro453Ser)
NCCAH samples (7)	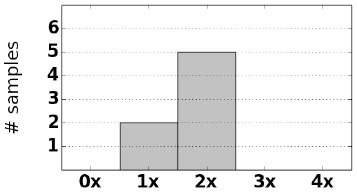	7 × CH5	2	g.655C>G
				g.1683G>T (p.Val281Leu)
				g.1689A>G (p.Met284Val)
CAH carriers (9)	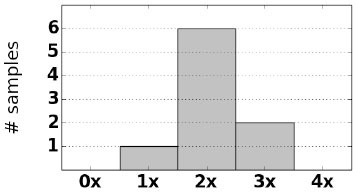	4 × CH5	1	g.655C>G
				g.1683G>T (p.Val281Leu)
				g.1994C>T (p.Gln318X)
				g.2444G>A (p.Arg408His)

For each sample we observe that CAH phenotype can be plausibly explained by either a complete loss of *CYP21A2*, a rearrangement that causes loss of gene function, or damaging small variants (Table 2). Of the samples with structurally normal *CYP21A2* genes, we found that those with CAH diagnosis had damaging variants that were either homozygous or heterozygous in *trans* configuration, thus the individuals can be inferred to have no fully functional copy of the gene. Each CAH carrier was found to have damaging heterozygous variants or heterozygous deletion of *CYP21A2*. In one particular carrier, damaging variants g.655C>G and g.2444G>A were observed in *cis* configuration, with the other copy of the gene unaffected.

**Figure 4:**
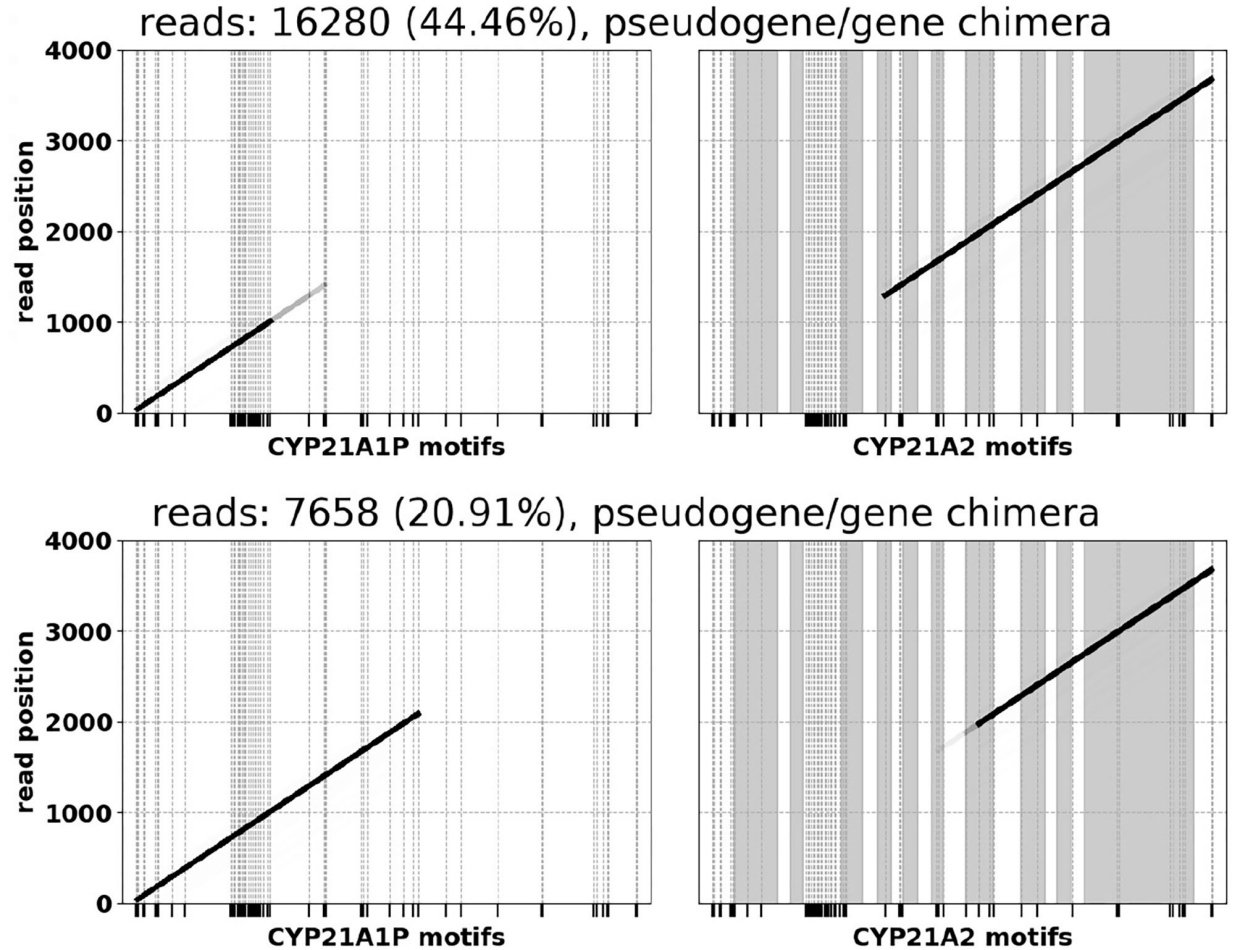
A1P/A2 chimeras observed in a SW CAH sample (CH2 on the top, CH7 on the bottom). Alignments in the left halves of the plots indicate sequences of pseudogene motifs, and the right halves correspond to gene motifs. *CYP21A2* exon regions are shaded and numbered.

In many samples we observe A1P/A2 chimeras resulting from a common ~ 30 kb deletion caused by misalignment during meiosis (Chen et al., 2011; Hannah-Shmouni et al., 2017). Following conventions in existing CAH literature, the chimeras are labeled based on the position where the A1P → A2 junction occurs (Hannah-Shmouni et al., 2017) [e.g., “CH2” (Lee et al., 2004) or “CH7” (Vrzalová et al., 2011)]. One of the salt-wasting CAH samples was observed to have three copies of A1P/A2 chimeras and no normal gene or pseudogene sequence (Figure 4). The 3x copy number was corroborated by variant allele fractions (VAF) in the long reads, where in an alignment of the chimeric reads heterozygous variants were identified at VAFs of ~ 33 and ~ 66%.

## 4. Discussion

From Figure 3, we see that PB-Motif is highly sensitive in detecting gene/pseudogene rearrangements for read error rates <8%. Within this range, nearly every read is correctly found to support the simulated structural variation. The performance drops off substantially at higher error rates, as it becomes less likely to encounter motif kmers unaffected by base-call errors. Variance in detection accuracy increases substantially above 7% error, which may also be a result of non-uniform motif density across the simulated *PMS2*/*PMS2CL* homology. Based on these results, we suggest that PB-Motif is best used with HiFi PacBio reads or other long reads that have been corrected to <8% error.

**Figure 5:**
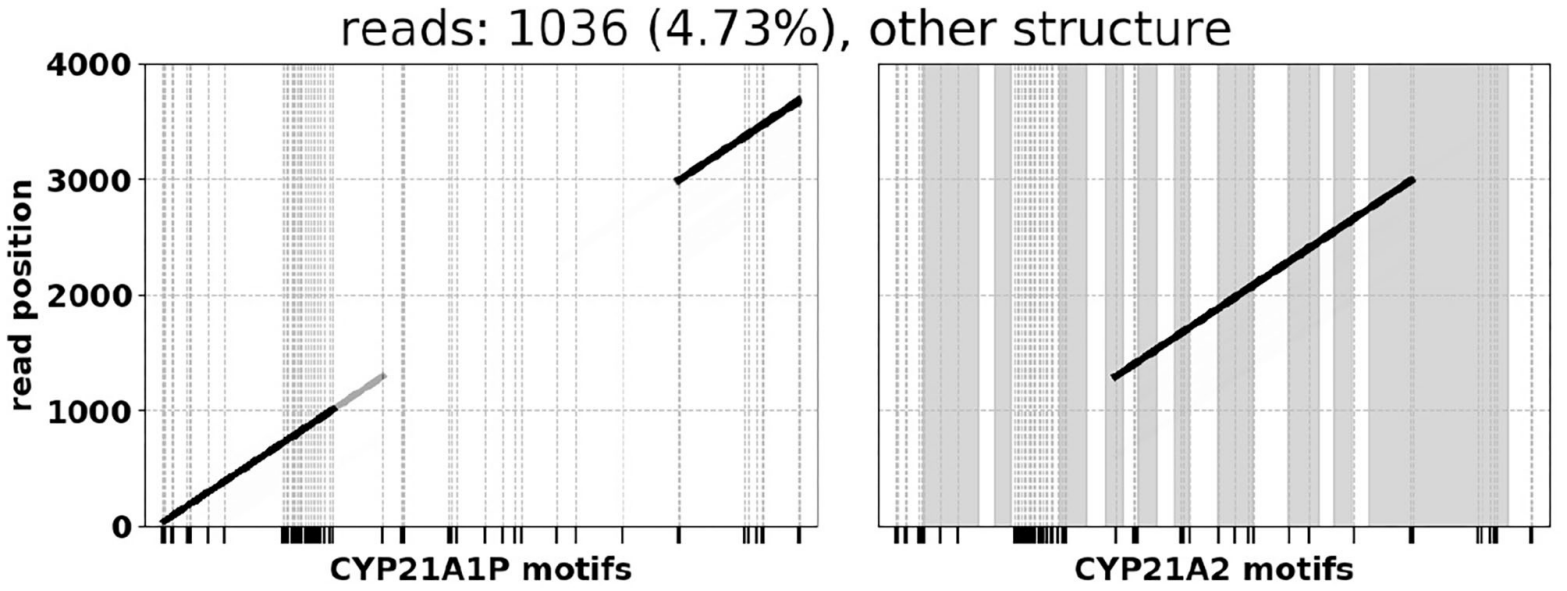
A1P → A2 → A1P rearrangement found in several samples.

In several of the CAH samples we observe what appears to be a migration of *CYP21A2* exons 8–10 into the pseudogene sequence (Figure 5). While numerous gene-derived variants have been reported in *CYP21A1P*, to our knowledge this particular intergenic recombination is not widely known, and could lead to false positives for methods that rely on distinguishing gene from pseudogene using *CYP21A2*-specific priming sites. It has been previously reported that the reference sequence for *CYP21A1P* may not be wholly representative of what is found in populations at large. Specifically, healthy individuals in German and Chinese populations have been found to have *CYP21A2*-like sequence within *CYP21A1P*, suggesting that what appears to be a structural rearrangement with respect to human reference hg38 may be more accurately characterized as a sequence belonging to an alternative reference assembly (Greene et al., 2014).

###  PCR Chimeras

On average we observe that ~ 5% of the reads per sample exhibit false-positive fusions of gene/pseudogene sequences. For example, in control samples known to contain solely normal *CYP21A2* and *CYP21A1P* sequences, these 5% of reads start in one region and end in the other, but otherwise pass all filtering criteria. We attribute this primarily to chimeric sequences formed during amplification (*in-vitro* PCR recombination), which is frequently observed when highly similar DNA are being amplified together (Smyth et al., 2010).

The breakpoints in these false-positives appear to be distributed randomly, and thus there are generally not enough reads for PB-Motif to report them as a rearrangement. However, in samples that do have chimeric gene/pseudogene rearrangements, these false positives slightly inflate the proportion of total reads that support chimeric patterns. Because of this, it is crucial to consider both variant allele frequencies in addition to read counts when estimating copy numbers of gene/pseudogene chimeras.

###  Applicability to Other Genes

PB-Motif is theoretically extensible to any pair of highly similar (but not identical) genomic regions. Such applications would require enumerating new motif kmers and designing a new capture strategy for the targeted gene/pseudogene regions.

To enumerate gene/pseudogene pairs to which PB-Motif might be applicable, we aligned protein-coding genes from RefSeq (release 90) with unprocessed pseudogenes from GENCODE (release 29). We restricted our attention to the pairs of regions that are > 1 kbp in size, > 90% homologous, and within 1Mbp of each other on the same chromosome. We applied these heuristics in order to identify regions that have the potential for exchanging damaging sequence content through gene conversion or crossover events, and are large enough such that they require long reads to genotype with high sensitivity. This exercise yielded 430 large, highly homologous gene/pseudogene pairs. By intersecting these 430 pairs with ClinVar (release 2018/09/30) we found 59 genes in which pathogenic variation has been observed, a subset of which is presented in Table 3 (full table in Supplementary Materials).

**Table 3:** Highly homologous gene/pseudogene regions to which our method might be applicable.

**Gene**	**Pseudogene**	**Largest homology**	**% identity**	**Clinical relevance**
*AGBL1*	*ADAMTS7P4*	1,476	94.72	Fuchs' corneal dystrophy
*ARMC4*	*ARMC4P1*	8,412	95.23	Ciliary dyskinesia, Kartagener syndrome
*BCR*	*BCRP1*	3,851	93.38	Chronic myelogenous leukemia
*CD46*	*CD46P1*	3,972	90.26	Atypical hemolytic uremic syndrome
*CEL*	*CELP*	3,218	97.02	Maturity-onset diabetes
*CYP21A2*	*CYP21A1P*	2,722	97.65%	21-OHD CAH
*CYP2B6*	*CYP2B7P*	5,336	92.77	Related to efavirenz response
*CYP2D6*	*CYP2D8P*	2,779	90.82	Related to the metabolism of multiple drugs
*DIS3L2*	*DIS3L2P1*	2,309	96.80	Nephroblastoma
*GBA*	*GBAP1*	1,024	97.66	Gaucher's disease, Parkinson's disease
*LPA*	*LPAL2*	1,670	93.59	Lipoprotein deficiency
*NCF1*	*NCF1C*	11,668	99.37	Chronic granulomatous disease
*PMS2*	*PMS2CL*	8,192	97.27	Lynch syndrome
*RNF216*	*RNF216P1*	7,078	96.00	Gordon Holmes syndrome
*STRC*	*STRCP1*	15,275	99.16	Non-syndromic hearing loss and deafness
*TNXB*	*TNXA*	2,373	99.49	Ehlers-Danlos syndrome, vesicoureteral reflux

Selectively amplified regions comparable in size to *CYP21A2* could be expected to yield HiFi long reads with very low error rates. Depending on sample preparation, genes with larger homologies such as *NCF1* or *STRC* might not yield polymerase reads long enough to be corrected to below 8% error. For these regions we speculate hybrid error correction approaches, e.g., using short reads sequenced from the same sample, might be necessary in order to apply PB-Motif. Alternately, for long reads with a distribution of different error rates, methods such as LongQC (Fukasawa et al., 2020) or SequelTools (Hufnagel et al., 2020) could possibly be used for filtering before running PB-Motif.

We note that some genes have multiple homologous pseudogenes, such as *BCR, CES1*, or *HERC2*, and that one possible extension of PB-Motif could be in identifying rearrangements between an arbitrary set of similar regions instead of between a single gene/pseudogene pair.

## Data Availability Statement

We have uploaded the simulated PacBio long reads used to assess our methodology to the Sequence Read Archive (SRA), under BioProject ID PRJNA736407.

## Ethics Statement

The studies involving human participants were reviewed and approved by Mayo Clinic IRB Application #: 21-002875. Feasibility of using PacBio long reads-based sequencing chemistry for genotyping of the CYP21A2 gene. Written informed consent for participation was not required for this study in accordance with the national legislation and the institutional requirements.

## Author Contributions

ZS designed and implemented PB-Motif. J-PK and RI contributed to experiment design. DM, BK, and SG conducted sample selection, sequencing, and clinical analysis. ZS and J-PK wrote the manuscript. All authors read and approved the final manuscript.

## Conflict of Interest

The authors declare that the research was conducted in the absence of any commercial or financial relationships that could be construed as a potential conflict of interest.

## Publisher's Note

All claims expressed in this article are solely those of the authors and do not necessarily represent those of their affiliated organizations, or those of the publisher, the editors and the reviewers. Any product that may be evaluated in this article, or claim that may be made by its manufacturer, is not guaranteed or endorsed by the publisher.
