# Supplementary material for: PB-Motif—A Method for Identifying Gene/Pseudogene Rearrangements With Long Reads: An Application to CYP21A2 Genotyping
Source: Front Genet. 2021 Jul 28;12:716586. doi: 10.3389/fgene.2021.716586 (PMC8355628; doi:10.3389/fgene.2021.716586)
Supplement: Supplementary file 1 [file Data_Sheet_1.PDF]

Supplementary figures:

- S.1: Overview of amplification primers and MLPA probes
- S.2: IGV screenshot of primer locations
- S.3: Example MLPA results
- S.4: Example Sanger results

S.1:

Amplification Primers and MLPA probe locations for CYP21A2

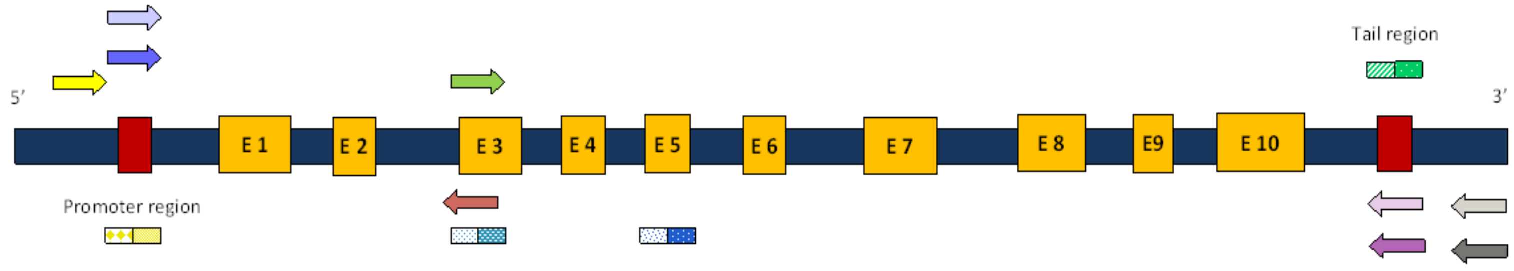

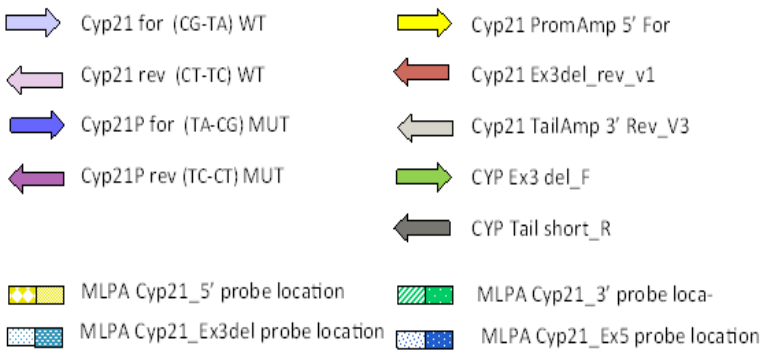

	32009351	32009354	32009375	32009385	Expected Gel	Expected MLPA
Normal A Tail	C	T	T	C	A	A
Hybrid A Tail	T	C	T	C	B	A
Normal B Tail	T	C	C	T	B	B
Hybrid B Tail	C	T	C	T	A	B

(Approximate sequencing amplicon locations.)

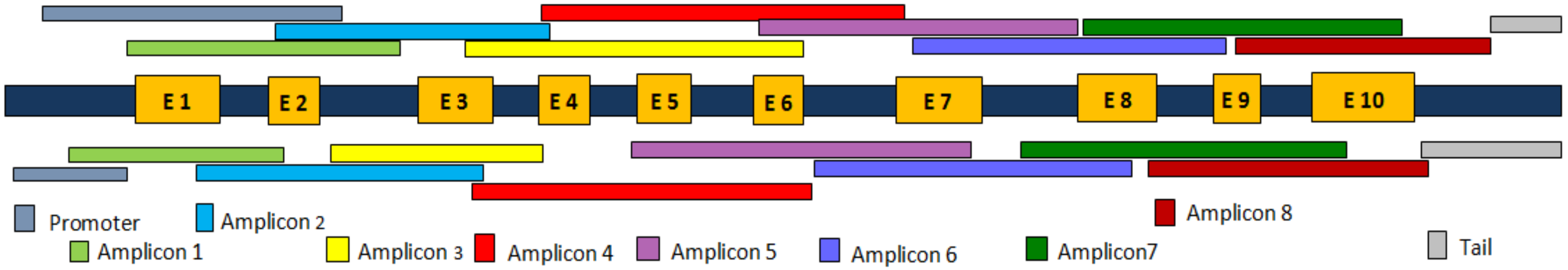

S.2: Universal primer overview

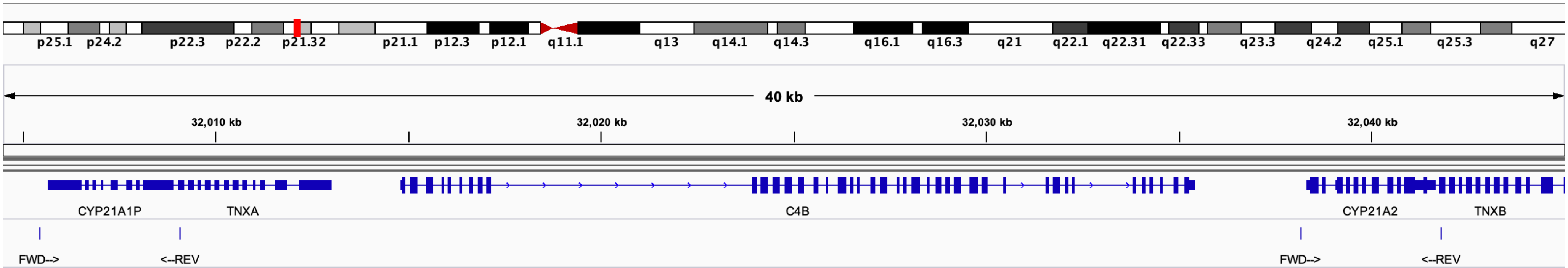

chr6:32005417-32005441	FWD--> 5'	CAGAAAGCTGACTCTGGATGCAGG
chr6:32009065-32009089	<--REV 3'	AACTGCCACTACGCCAACCTCAAC
chr6:32038152-32038176	FWD--> 5'	CAGAAAGCTGACTCTGGATGCAGG
chr6:32041800-32041824	<--REV 3'	AACTGCCACTACGCCAACCTCAAC

S.3: Example MLPA results

MLPA Analysis Report - SoftGenetics	
**Software:** GeneMarker V2.4.0	**Analysis Type:** MLPA
**Project:** Untitled	**Compare Type:** MLPA Ratio
**Technician:**	**Normalization By:** Population Normalization (Adjusted)
**Report Time:** 03/22/2017 - 15:22:51	**Quantification By:** Peak Height
**Panel:** LX10007162403	**Classification:** Loss < 0.80 <= Equivalent <= 1.25 < Gain
**Control:** Synthetic Control Sample	**Report Value Type:** Peak Ratio
**Synthetic Used:** B1_F1 /// H1_F2 /// C2_F3	

	Probe Name	Bin Size	B2_17-FDZ96
1	**01-BCAR**	50.8	0.960
2	**02-NPC17**	55.4	1.028
3	**03-HIRA**	60.0	0.998
4	**04-TNFRSF7**	64.6	1.014
5	05-ZFX	69.2	0.950 ✓
6	06-Cyp21_5'(N)	73.8	1.271 3
7	07-Cyp21_3'(N)	78.4	1.238 3
8	08-Cyp21_5'(P)	83.0	0.686 1
9	09-Cyp21_3'(P)	87.6	0.627 1
10	10-Cyp21_Ex3del	92.2	0.557 1
11	11-Cyp21_Ex5	96.8	0.967 2

→ 3 copies normal gene sequence

→ 1 copy normal pseudogene sequence

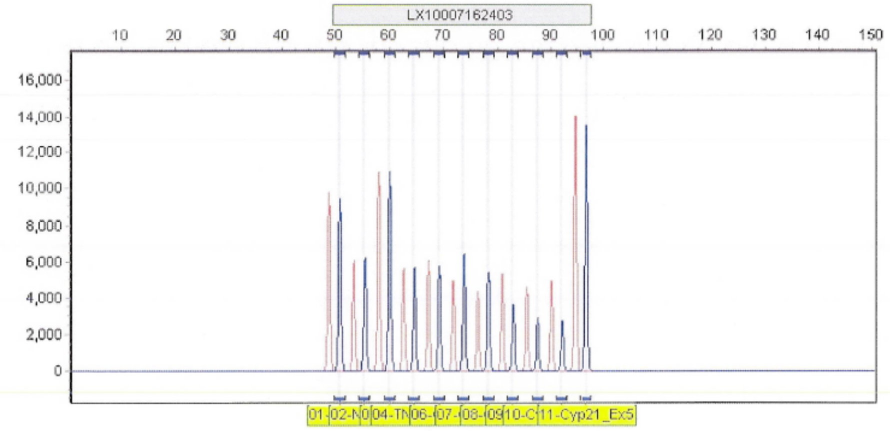

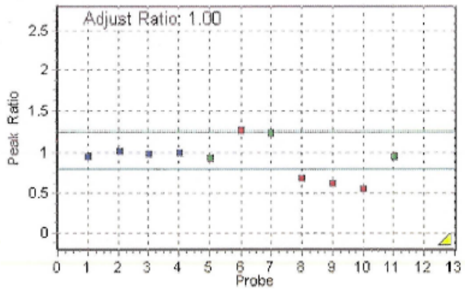

Screenshot of GeneMarker results of MLPA on one of the SW CAH samples. From the relative amplitudes of each probe we infer that this sample has 3 copies of CYP21A2, 1 copy of CYP21A1P, and no copies of any chimeras.

S.4: Example Sanger sequencing to validate Q319X mutation

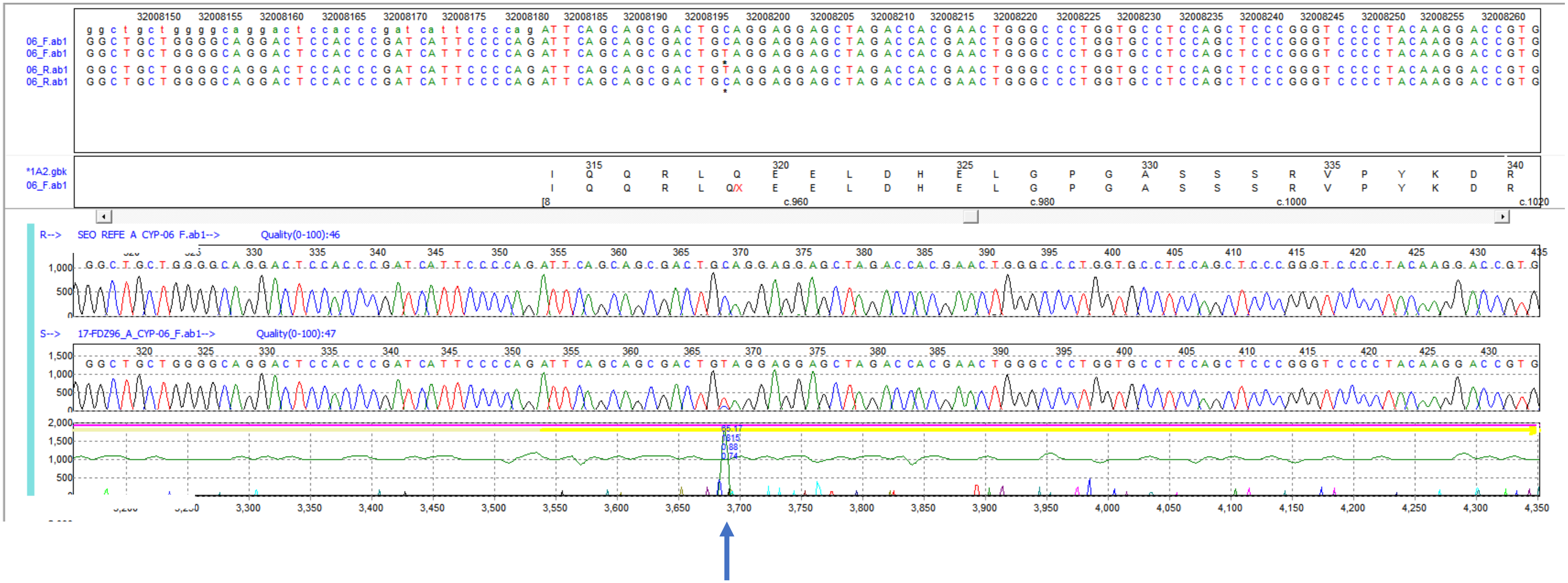

Sanger sequencing of a SW CAH sample with heterozygous g.1994C>T (p.Q319X) mutation.
